# Liver abscess as a complication of acute Lyme disease: a case report

**DOI:** 10.3389/fmed.2026.1708450

**Published:** 2026-03-04

**Authors:** Sam T. Donta, Devin A. McManus, Kenneth L. Caswell

**Affiliations:** 1 Department of Medicine, Falmouth Hospital, Falmouth, MA, United States; 2 Department of Radiology, Falmouth Hospital, Falmouth, MA, United States

**Keywords:** liver abscess, liver function tests, liver ultrasound, Lyme disease, Lyme Western blots

## Abstract

This is a report of a patient who presented with acute Lyme disease and was found to have a liver abscess as part of evaluation of abnormal liver test abnormalities. He had had a prior ultrasound 8 months previously as part of evaluation for a potential abdominal aneurysm and no abscess had been noted at that time. No other cause for the abscess was found, and the abscess resolved with coincident antibiotic treatment for his Lyme disease.

## Case report

In May of 2024, a 56 years old man presented with fever, extreme fatigue, headache, arthralgias, thigh pains, and chills of 4 days duration. He had had prior tick bites and Lyme disease, as he frequently walked in the woods with his dog. And those episodes had resolved with antibiotic treatment. The remainder of his review of symptoms and his physical examination were unremarkable. Ongoing medications included atorvastatin, valsartan, and rizatriptan. The patient was started on doxycycline and further tests ordered.

The results of routine blood work were negative with the exception of an elevated WBC of 14.9k. Testing for tick-borne illnesses by PCR and tick-specific antibodies were negative except for Lyme disease. A Lyme antibody screen was positive at 1.99 and Western blots revealed an IgM reaction to the 23kd protein, and IgG reactions to the 18kd, 23kd, 39kd, 41kd, 58kd, and 66kd proteins.

After 10 days of treatment with doxycycline, he reported some improvement, but had persistent fevers, general lassitude, but no joint pains or other symptoms. His doxycycline treatment was extended to 3 weeks. He felt well for 3 weeks afterward, had 2 additional tick bites in the interim, then relapsing fever, and felt sick. The results of blood tests at that time revealed a WBC of 13.4k, an ESR of 128 mm/hr, a CRP of 244 mg/L, and elevated liver function tests (ALT-68, AST-62, Alk Phos-654). A Lyme antibody screen was 1.72. Lyme Western blots revealed the same reactions as before except for the absence of positivity for the IgG 58kd and 66kd bands. Retesting for other tick-borne diseases again revealed no positive PCRs or tick-specific antibodies. The results of blood cultures were negative. Cefuroxime was prescribed to take for 2 weeks. The results of blood tests a week after the start of cefuroxime revealed a normal WBC of 7.0k, an ESR of 70/mm, and CRP of 72.1. Cefuroxime was continued, and a 10 days course of empiric treatment of azithromycin and atovaquone for possible babesiosis was added.

**FIGURE 1:**
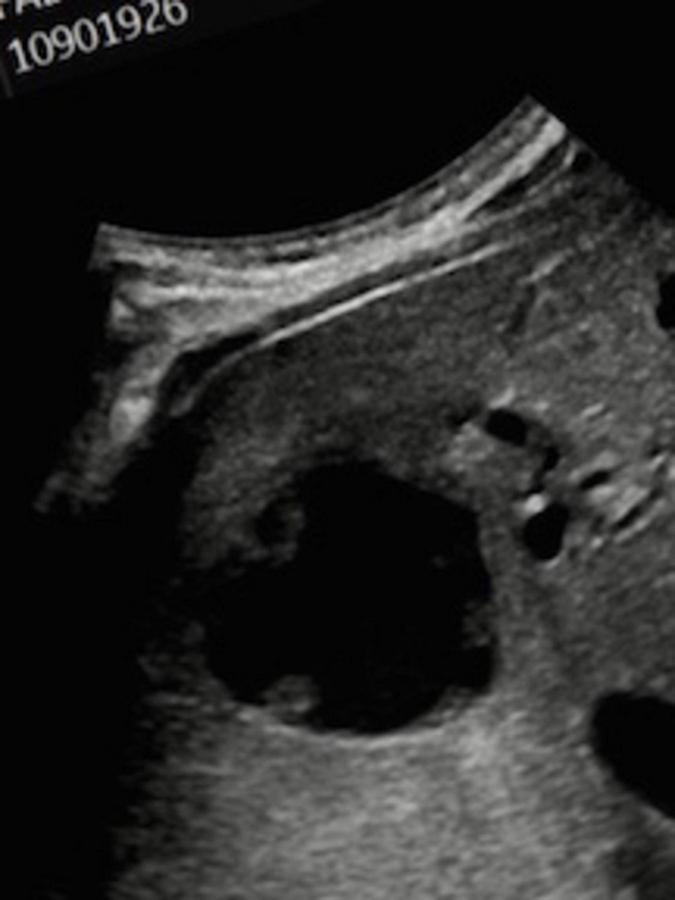
Ultrasound of liver showing an abscess. There is a mildly irregular, predominantly cystic lesion within the central hepatic parenchyma measuring 4.9 cm in largest dimension, new since a prior comparison ultrasound performed 8 months ago.

Because of the elevated liver function tests, an ultrasound of his abdomen was ordered, and an oval irregular predominately cystic lesion within the right lobe of the liver, 4.9 cm in its largest dimension was seen. The lesion was mildly lobulated with ill-defined margins (Figure 1). There were no other intra-abdominal abnormalities noted. A MRI 2 weeks later showed a 75% decrease in the cystic structure, suggesting a resolving liver abscess, now measuring 2.8 cm in its largest dimension (figure not shown). The results of blood tests at that time in early August revealed a normal WBC of 6.4k, a normal ESR of 2/mm, a normal CRP of 4.3 mg/L, and a normal ALT. The results of all WBC, ESR, CRP, and LFT blood tests are shown in Table 1. Additional testing for *Entamoeba histolytica* was negative.

**TABLE 1:** Blood test results.

Date	WBC	ESR	CRP	ALT	AST	ALK	BIL
		mm/hr	mg/L	U/L	U/L	U/L	mg/dl
5/14/24	14.9k						
7/2/24	13.4k	128	244	68	62	654	2.0
7/9/24	9.6k	70	72.1				
8/8/24	6.4k	2	4.3	46	36	149	1.6
9/3/24				24	25	122	1.1
11/6//24				24			

**FIGURE 2:**
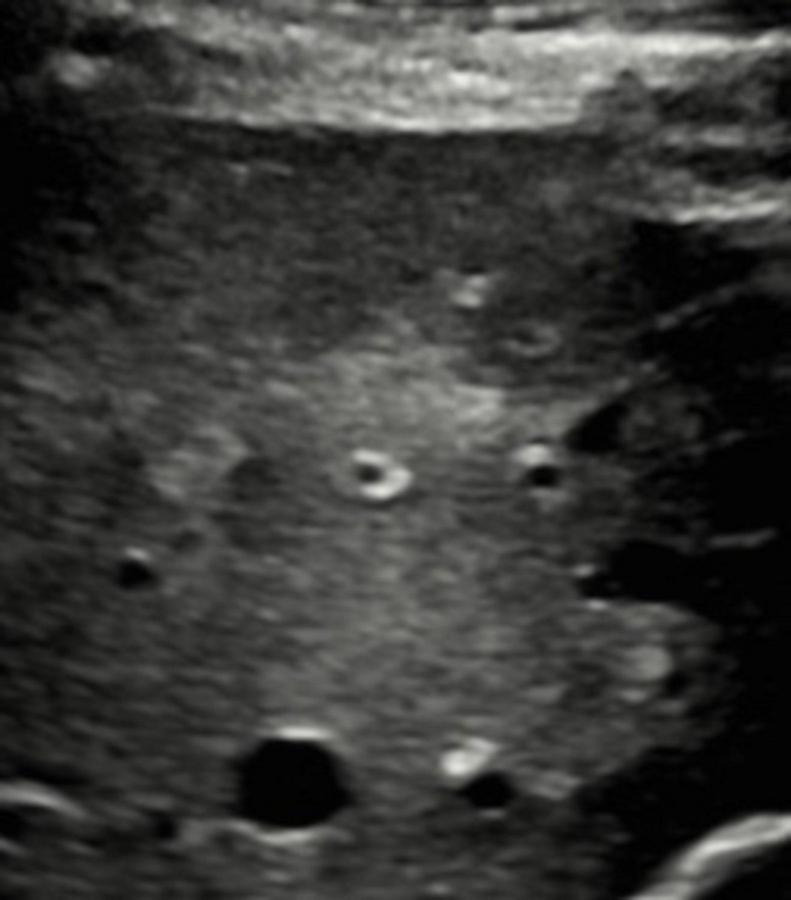
Follow up ultrasound of the liver no longer showing abscess. Four months follow up right upper quadrant ultrasound demonstrates interval resolution of the central hepatic lesion.

After completion of his antibiotic treatments, he felt well, had no further symptoms and remained well when last seen in November. Repeat ultrasound of his liver at that time revealed no evidence of the prior liver lesion (Figure 2).

## Discussion

Liver abnormalities in the form of elevated liver function tests with acute Lyme disease are not unusual (1–4). What has not been previously reported, is an abscess associated with acute Lyme disease. The patient had an ultrasound of his abdomen in December of 2023 looking for a potential abdominal aneurysm, and no lesions were noted in his liver at that time. Thus, without any obvious cause of the liver lesion other than Lyme disease, and with resolution of the lesion coincident with appropriate antibiotic treatment, it seems reasonable to conclude that Lyme disease was the likely cause of the abscess. An otherwise non-descript cyst of the liver would not be an explanation of his ultrasound findings, given that it had not been previously noted, the large size of the cyst, and it’s coincident resolution with antibiotic treatment. Other causative considerations, such as a gram-negative bacterium (e.g., *Klebsiella* sp.), without more severe symptomatology and positive blood-culture results would be unlikely. More definitive evidence that the liver lesion was an abscess caused by the Lyme spirochete would have involved visualization, PCR detection, or culture of the spirochetes from the liver abscess. This case report illustrates the importance of the potential for liver abscess in patients with Lyme disease.

## Data Availability

The raw data supporting the conclusions of this article will be made available by the authors, without undue reservation.

## References

[1] Kazakoff M Sinusas K Macchia C. Liver function test abnormalities in early Lyme disease. *Arch Fam Med.* (1993) 4:409–13. 10.1001/archfami.2.4.409 8130920

[2] Nadelman R Nowakowski J Forseter G Goldberg N Bittker S Cooper D The clinical spectrum of early Lyme borreliosis in patients with culture-confirmed erythema migrans. *Am J Med.* (1996) 5:502–8. 10.1016/s0002-9343(95)99915-9 8644761

[3] Horowitz H Dworkin B Forseter G Nadelman R Connolly C Luciano B Liver function in early Lyme disease. *Hepatology.* (1996) 6:1412–7. 10.1002/hep.510230617 8675158

[4] Nadeem M Tafader A Markley J Bajaj J. Liver manifestations of tick-borne diseases. *Clin Liver Dis.* (2023) 4:111–6. 10.1097/CLD.0000000000000025 37197220 PMC10184991

